# Progress in Immunoassays of Toxic Alkaloids in Plant-Derived Medicines: A Review

**DOI:** 10.3390/toxins14030165

**Published:** 2022-02-23

**Authors:** Zhenhui Ren, Huixia Zhang, Zile Wang, Xin Chen, Liu Yang, Haiyang Jiang

**Affiliations:** Department of Veterinary Pharmacology and Toxicology, College of Veterinary Medicine, China Agricultural University, Beijing 100193, China; sxfyrzh@163.com (Z.R.); zhanghuixia2008@163.com (H.Z.); wangzile2017@163.com (Z.W.); b20183050402@cau.edu.cn (X.C.); 2016305010314@cau.edu.cn (L.Y.)

**Keywords:** toxic alkaloid, plant-derived medicine, hapten design, antibody, immunoassay

## Abstract

Plants are the cradle of the traditional medicine system, assuaging human or animal diseases, and promoting health for thousands of years. However, many plant-derived medicines contain toxic alkaloids of varying degrees of toxicity that pose a direct or indirect threat to human and animal health through accidental ingestion, misuse of plant materials, or through the food chain. Thus, rapid, easy, and sensitive methods are needed to effectively screen these toxic alkaloids to guarantee the safety of plant-derived medicines. Antibodies, due to their inherent specificity and high affinity, have been used as a variety of analytical tools and techniques. This review describes the antigen synthesis and antibody preparation of the common toxic alkaloids in plant-derived medicines and discusses the advances of antibody-based immunoassays in the screening and detection of toxic alkaloids in plants or other related matrices. Finally, the limitations and prospects of immunoassays for toxic alkaloids are discussed.

## 1. Introduction

In clinical medication practice, the use of plant-derived medicines in the treatment of human or animal diseases, such as inflammation and cancer, has been documented for over 5000 years [1,2,3,4]. Owing to the outstanding advantages, such as the nature of the original compounds, the functionality of a lot of active ingredients, and the low cost of a wide range of sources, plant-derived medicines are becoming more and more popular with consumers worldwide. In the current era of widespread antibiotic resistance, plant-derived medicines such as garlic have remarkably persisted over time and continue to be used today for indications similar to those described historically, which can be used as alternative antibiotic resources [5]. In addition, plant-derived medicines as functional foods have made a significant contribution to human or animal health [6]. It has been estimated by the World Health Organization that up to 80% of the world’s population, mostly in developing countries, rely on plant medicines for primary health care [7,8,9,10]. They are classified as complementary and alternative medicines and are regulated by the Therapeutic Goods Administration in Australia and the Dietary Health and Supplement Education Act under the Food and Drug Administration in the USA [11,12,13,14,15]. As feed additives, plant-derived medicines play an important role in promoting growth, anti-inflammatory, increasing immunity, and other functions [16,17,18,19,20].

Alkaloids are a very large and chemically inhomogeneous group of nitrogen-containing compounds in plant-derived medicines, which are water-soluble under acidic conditions and lipid-soluble under neutral and basic conditions [21,22]. They have a wide range of pharmacological activities, such as anti-cancer, cardiotonic, analgesic, lower blood pressure, anti-bacterial, and anti-inflammatory [23,24,25,26,27,28]. However, some alkaloids not only have clinical value but also have different degrees of toxicity. Poisoning of people and livestock caused by improper treatment, accidental ingestion, homicide, or accident frequently occurs and seriously limits the safety of clinical use of plant-derived medicines [29]. Morphine, codeine, and cocaine, the first isolated secondary metabolites, have been transformed into important drugs due to the effect of local anesthesia [30,31]. Yet people’s abuse or long-term use of these alkaloids can cause an irregular heart rate and myocardial infarction and disrupt the balance of various physiological tissues in the body. Pyrrolizidine alkaloids are a class of toxic alkaloids with hepatotoxicity, derived from plant families such as *Asteraceae*, *Boraginaceae*, or *Fabaceae* [32,33]. Humans ingested these compounds by ingesting contaminated herbal medicines or teas [33,34]. Incidents of aconitine poisoning caused by improper plant handling or improper ingestion also frequently occur [35,36,37,38]. In addition, toxic alkaloids can indirectly damage human and animal health through the food chain. For instance, atropine is illegally injected into pigs and causes them to drink a lot of water and gain weight in the short term. The substance remains in pork and enters the human body through the food chain, resulting in coma, convulsions, and other toxic phenomena [39]. The related information of the common toxic alkaloids in plant-derived medicines reported in the literature, including their structure, classification, CAS registry number (a unique numeric identifier that is designated only one substance), source, toxicity, and median lethal dose (LD_50_) of mice and rats, are summarized in Table 1. These toxic alkaloids have various structures, low toxic dose, rapid metabolism in vivo, and no specific clinical manifestations after the poisoning, which directly increase the difficulty of screening and analyzing the related target substances in complex biological systems. At present, there is still a lack of internationally recognized limit specifications and quantitative detection standards for these harmful substances.

A large number of analytical methods based on different analytical techniques have been developed for the determination of toxic alkaloids in plant-derived medicines or other related matrices, e.g., thin-layer chromatography (TLC) [40,41], liquid chromatography-mass spectrometry (LC-MS) [42,43,44,45], gas chromatography-mass spectrometry (GC-MS) [46,47], and ultraviolet spectrophotometry [48]. However, these instrumental methods for monitoring toxic alkaloids have certain disadvantages and limitations, such as being time-consuming, having expensive instruments, and high requirements for the experimental instruments, thus, these methods lack field applicability. More importantly, different toxic alkaloids of the same species are rich in structure and abundant in quantity, and there are isomers, which need to be detected with standard substances as the control. Therefore, it is an urgent task to develop a cheap and reliable method for the effective on-site monitoring of toxic alkaloids in plant-derived medicines or other matrices.

**Figure 1:**
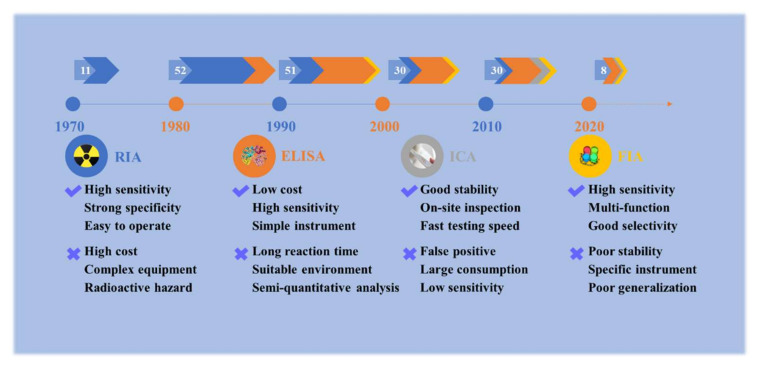
Development and scientific articles of immunoassays for plant-derived toxic alkaloids. The numbers in white represent the number of articles per decade. RIA: radioimmunoassay; ELISA: enzyme-linked immunosorbent assay; ICA: immune-chromatography assay; FIA: fluoroimmunoassay.

In the late 1960s, the introduction of labeled antibody technology brought many benefits to medical and clinical diagnosis technology [49]. The immunoassay is a rapid detection method based on the specific reaction of antigen and antibody, which amplifies the reaction signal of labeled antigens or antibodies by enzymes, radioactive elements, colloidal gold, or other available labeling materials. They have been routinely used in a wide spectrum of compounds such as proteins, pesticides, veterinary drugs, biomarkers, and heavy metals. The main advantages of immunoassays are that they are inexpensive, suitable for high throughput, and do not require high-tech equipment or professional technicians. Thus, they could be conducted much more economically than the high-tech instrumental methods, and they are continuously expanding, especially in the development of more sensitive, rapid, specific, and robust analytical methods. For toxic alkaloids, literature about antibodies or immunoassays from 1 January 1970 to 31 January 2022 were retrieved from PubMed (http://pubmed.cn/, accessed on 31 January 2022) and Web of Science (http://www.isiknowledge.com/, accessed on 31 January 2022) with the MeSH (Medical Subject Headings) word “aconitine” (for example) and “antibody” or “immunoassay” without language restriction. A total of 453 articles were retrieved. After excluding repetitive articles and literature based on commercial antibodies, 182 articles were used for data extraction and analysis. The application of immunoassay in toxic alkaloids began in the 1970s, and it has mainly gone through four stages: radioimmunoassay (RIA), enzyme-linked immunosorbent assay (ELISA), immunochromatography assay (ICA), and fluoroimmunoassay (FIA), as shown in Figure 1. Yet, it is only widely used in highly toxic or abused toxic alkaloids, such as aconitine, codeine, morphine, etc., and less used in other toxic alkaloids with few reports of poisoning incidents. Furthermore, the structure of toxic alkaloids is complex and the cost of obtaining them from plants is high. Antibody preparation based on the traditional trial-and-error method requires a large number of standard substances, and it is difficult to obtain ideal broad-spectrum antibodies, so the development of immunoassay of toxic alkaloids is slow. This review focuses on the recent advances in the field of immunoassays for the rapid detection of toxic alkaloids, including antigen preparation, antibody production, and various types of methods reported in the literature. Finally, the limitations and prospects for the immunoassay of toxic alkaloids in plant-derived medicines are also discussed.

## 2. Synthesis and Identification of Antigen

### 2.1. Selection of Hapten

Alkaloids, an important class of small-molecule natural compounds (molecule weight < 1000 Da), usually need to bind to large molecules to induce immune response [50]. In general, if the analyte contains active groups such as amino, carboxyl, hydroxyl, or sulfhydryl, these groups could be directly linked to the carrier protein. Tanaka et al. [51] successfully prepared an antigen that directly coupled the hydroxyl group in the structure of solamargine with bovine serum albumin (BSA). To avoid masking the characteristic structure of small molecules by proteins, a spacer arm is usually introduced, which is generally a non-polar carbon chain, and should not contain other structures with high immune activity, such as benzene rings, heterocycles, halogen atoms, etc. Kiko et al. [52] prepared 3-succinylaconitine by succinylation at the C-3 hydroxyl position of aconitine. The same method has also been used to prepare haptens such as camptothecin [53], morphine [54], retrorsine [55], and tropane [56]. Wang et al. [57] prepared a hapten of methamphetamine by introducing a carboxyl group on the second amino group. Compounds that react with the active groups can also be replaced by bromoacetic acid [58], aminobutyric acid [59], or other substances, with appropriate chain length [57].

However, if there is no active group or the active group greatly influences the specificity and polarity of the compound, haptens for artificial antigen synthesis need to be designed or modified from scratch. Sakurai et al. [60] designed two haptens of cocaine for the preparation of antibodies. The analog incorporated an amido linker functionality in place of the carbomethoxy group at C-2 and benzoyl amino-substituted for the benzyloxy group at C-3 of the cocaine framework. Kikuchi et al. [61] synthesized scopolamine from scopolamine, exposing the amino group at the active site and synthesizing a hapten containing a carboxyl group at the terminal for protein coupling.

Haptens can also be designed by the target’s precursors or metabolites. Sakamoto et al. [62] synthesized a hapten of monocrotaline by using retronecine (a hydrolysate of monocrotaline). Heroin is a highly toxic alkaloid derived from opium. It is first metabolized into monoacetylmorphine (MAM), and MAM is then further converted into morphine [63]. Gandhi et al. [64] synthesized an acidic derivative of MAM and eventually induced antibodies that simultaneously recognize heroin, MAM, and morphine because they have the same common structure. Wang et al. [56] selected the simplest compound with the structure of tropine ring from 12 kinds of atropine alkaloids with similar structures and their structural analogs for hapten design and finally obtained an antibody that can cross-recognize seven kinds of atropine alkaloids.

Designation at different sites in the compound also exposes different fragments of the molecule to immune B cell epitopes, generating antibodies with different recognition properties. Yan et al. [53] designed two haptens based on B- and E-ring modifications to investigate the effects of haptens’ analogs on the sensitivity of immunization. One case is to introduce an active carboxyl group at the C-7 site, and the other case is to introduce a four-carbon spacer arm containing a carboxyl group to connect to the hydroxyl group at the C-20 site. The electron distribution analysis showed that the latter hapten maintained the parent ring structure of camptothecin, and the second haptens were used to obtain monoclonal antibodies with high sensitivity and high titer. Therefore, the stability of haptens is also one of the key factors that cannot be ignored in the process. Trofimov et al. [65] obtained two morphine derivatives, 6-hemisuccinyl and 3-O-carboxymethyl esters, and their antibodies. The results showed that antibodies derived from 3-O-carboxymethyl interacted with morphine more effectively than 6-hemisuccinyl.

Computer-aided molecular modeling is an effective strategy, which can evaluate and select the designed haptens from the aspects of molecular configuration and electronic properties, and ultimately improve the possibility of obtaining the target antibodies. Now, this method has only been preliminarily applied to atropine [56] and pyrrolizidine alkaloids [55], and other related studies have not been reported. The structures of the toxic alkaloids in plant-derived medicines are complex and varied, but the same species has the same parent structure and properties. If the efficient technique can be used to produce monoclonal antibodies (mAbs) of other toxic alkaloids, it is believed that more specific or broad-spectrum target antibodies can be obtained, reducing the waste of laboratory animals and trial-and-error processes.

### 2.2. Coupling and Identification of Hapten

Currently, the carriers for hapten coupling are mainly proteins, including BSA, ovalbumin (OVA), keyhole limpet hemocyanin (KLH), human serum albumin (HSA), etc. BSA is most commonly used in the toxic alkaloids field due to its stable physicochemical properties, non- dewaterability, low cost, and easy availability, as well as its high lysine content (i.e., high coupling density of hapten and carrier) [52,53,57,58,64,66,67,68,69,70,71,72,73,74]. Similarly, HSA also has a large amount of lysine and more free amino acids, which are widely used [59,75]. KLH is also commonly used as an immunogen protein [53,70,76,77]. It is more expensive than BSA and has poor water solubility, but it has higher molecular weight and better immunogenicity than BSA. Yan et al. screened various carrier proteins to select the most suitable immunogen for the production of mAbs against camptothecin, and KLH showed better immunogenicity than BSA. OVA is characterized by poor solubility, instability, and variability, and contains fewer active groups than BSA, but it is often used as an unrelated carrier protein for antibody screening and immunoassays [53,61,64,73,78,79].

**Figure 2:**
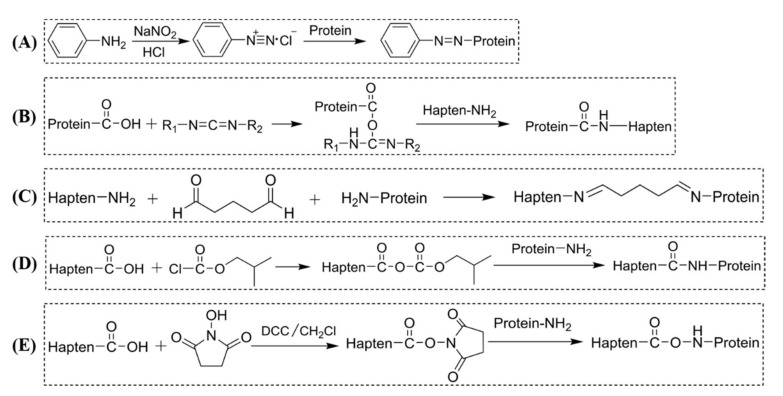
Common methods for conjugating hapten to the protein of toxic alkaloids. (**A**) Diazo method; (**B**) carbodiimide method; (**C**) glutaric dialdehyde method; (**D**) mixed anhydride method; (**E**) active ester method.

Modified toxic alkaloids and their derivatives are haptens usually carrying carboxyl or amino groups, but a few contain other groups such as aldehyde. The carbodiimide method (Figure 2B) [73,80,81], mixed anhydride method (Figure 2D) [53,82], and active ester method (Figure 2E) [31,52,53,78,83] are the most widespread and universal coupling methods used for hapten containing carboxyl groups. The glutaric dialdehyde methods (Figure 2C) [72,84] are often used for haptens containing amino groups. Cibotti et al. [84] modified the structure of VLB and introduced amino groups at the C-3 position, and successfully conjugated with BSA by the glutaric dialdehyde method to form an immunogen VLB_C3_-BSA. Antigen VLB_C17_-BSA was also synthesized by introducing BSA at active aromatic hydrogen C17 in VLB by the Mannich reaction. Comparing the monoclonal antibodies prepared by the two immunogens, the latter antibody is more susceptible to structural changes and has a higher specificity for VLB. Besides, diazo methods (Figure 2A) are commonly used for reactions containing aromatic amine groups, e.g., cocaine [85] and atropine [59]. Stammel et al. [86] reported a method of reductive amination, which is a very effective method for coupling small molecules bearing an amino group to carrier proteins. For haptens containing aldehyde groups, Ishiyama et al. [87] and Putalun et al. [77] used NAIO_4_ to oxidize the adjacent dihydroxyl groups on the C-3 sugars of solamargine into aldehyde groups. Then it was linked with BSA and KLH to synthesize immune antigen and coated antigen. The connection position and method of the hapten and the carrier protein are important factors that affect the specificity of the resultant antibody. Pontikis et al. (1980) synthesized three haptens conjugated to BSA at different coupling sites on three rings of colchicine. Antibodies exhibited variable cross-reactivity (CR) to the metabolites and structural analogs of colchicine, depending on the site where the colchicine is coupled to the protein carrier. The same work also demonstrated that attaching haptens to different sites, exposing different surfaces opposite the small molecule antigen junction, has a significant effect on the specificity of antibodies [72].

The methods commonly used for the identification of toxic alkaloid-carrier protein conjugates are ultraviolet and visible spectrophotometry (UV-Vis), sodium dodecyl sulfate polyacrylamide gel electrophoresis (SDS-PAGE), and matrix-assisted laser desorption/ionization time of flight mass spectrometry (MALDI-TOF-MS) [52,53,57,81,88,89]. In general, MALDI-TOF-MS is the ideal analytical method for the determination of coupling due to its high sensitivity, small sample quantity, and accurate and reliable results, and the coupling rate can also be measured [55,56,62,90]. Kido et al. used MALDI-TOF-MS to accurately determine the molecular weight range of the conjugate of aconitine derivatives-BSA between 68,700 and 72,800, and the mean fractionation size of the conjugate was 70,500. The BSA fractionation amount was 66,000 and the molecular weight of the aconitine derivative was 4500. The calculated coupling rate ranged from 4 to 9 with an average of 6, which could be used for immunity. However, it is inconvenient for general laboratories because of its complicated operation, high technical requirements, and high cost.

## 3. Types and Preparation of Antibodies

The research on toxic alkaloids’ antibodies has experienced three stages: polyclonal antibodies (pAbs), monoclonal antibodies (mAbs), and genetic engineering antibodies. The first two antibodies are usually referred to as traditional antibodies, and the third one is called novel antibodies. Through traditional hybridoma technology and display technology (such as phage display, mammalian cell display, and polysome display), antibodies can be successfully obtained.

In the process of an antibody preparation, the effective immunogen and the body’s reaction to the antigen stimulus are the key factors to evaluate whether the antibody can be produced. The adjuvant is a good auxiliary reagent, which may enhance the long-term release of the antigen by functioning as a depot, and may also act as non-specific mediators of immune cell function by stimulating or modulating immune cells. Complete Freund’s adjuvant and incomplete Freund’s adjuvants are often used in the preparation of antibodies against toxic alkaloids [52,55]. A recent study showed that chiral nanoparticles as immune adjuvants could enhance immune response, which provides new ideas for the preparation of toxic alkaloid antibodies [91]. Compared with traditional adjuvants, nanoparticle materials have more kinds and higher safety, and have the potential for a new generation of efficient adjuvants.

Based on the literature analysis, the synthesis of antigens and the preparation of antibodies are summarized in Table 2.

### 3.1. Polyclonal Antibody

PAbs are antibodies derived from different B cell sources that can recognize multiple epitopes on an antigen. These antibodies are usually produced by immunizing larger mammals, such as rabbits or goats, to collect a greater volume of serum, and the production cost is inexpensive. Polyclonal antibodies generally have a higher affinity than monoclonal antibodies. In 1970, the polyclonal antibody of morphine was first reported, and the technique began to be widely used in the quantitative analysis of other toxic alkaloids [54]. Xu et al. (2015) synthesized aconitine immunogen by the active ester method and prepared polyclonal antibody against aconitine by immunizing New Zealand white rabbits with a serum titer of 1:1,280,000. Yuan et al. (2016) synthesized the immunogen of benzoylmesaconitine using the same method as above. The detection limit of the polyclonal antibody was 139 pg, which could be used to identify monoester and diester alkaloids in aconitum plants. Stamme et al. (1993) immunized rabbits to obtain highly specific and sensitive polyclonal antibodies with a titer of 1:1700, which can be used to detect salsolidine in tissues and blood.

### 3.2. Monoclonal Antibody

MAbs are identical immunoglobulins, generated from a single B cell clone. These antibodies recognize unique epitopes, or binding sites, on a single antigen. The derivation from a single B cell clone and subsequent targeting of a single epitope is the difference between monoclonal antibodies and polyclonal antibodies. Currently, the preparation and application of monoclonal antibodies in toxic alkaloids are also in a stage of rapid development. Although monoclonal antibodies based on hybridoma technology have obvious disadvantages such as complex preparation, long cycle, and high cost, they are available in unlimited quantities and can be used in commercial immunoassay products. Kikuchi et al. (1991) prepared a highly sensitive monoclonal antibody with a detection limit of 0.2 ng/mL by fusing NS1 myeloma cells with spleen cells of BALB/c mice immunized with scopolamine. The monoclonal antibody to the scopolamine cross-reacts with methscopolamine and Scopolamine Butylbromide, but not with atropine, tropic acid, 6β-hydroxyhyoscyamine, and 7β-hydroxyhyoscyamine. Kim et al. (2004) prepared the monoclonal antibody against berberine which can identify isoquinoline alkaloids in herbal medicines.

### 3.3. Genetically Engineered Antibody

The genetically engineered antibody is recombinant and cloned into an expression vector, expressed in an appropriate host, and folded into a functional antibody molecule using genetic engineering technology. It has the characteristics of small molecular weight, strong plasticity, low cost, and mass production without animal immunity. It mainly includes chimeric antibodies, humanized antibodies, complete human antibodies, single-chain antibodies fragments, bispecific antibodies, and so on. Currently, only Brennan et al. [100] have reported a single-chain antigen-binding protein (scFv) that can recognize morphine and has been obtained and successfully applied to real samples’ analysis in saliva. In the rapid development of gene-engineered antibodies, they will inevitably become the new favorite in the detection of toxic alkaloids.

## 4. Detection Assay

**Figure 3:**
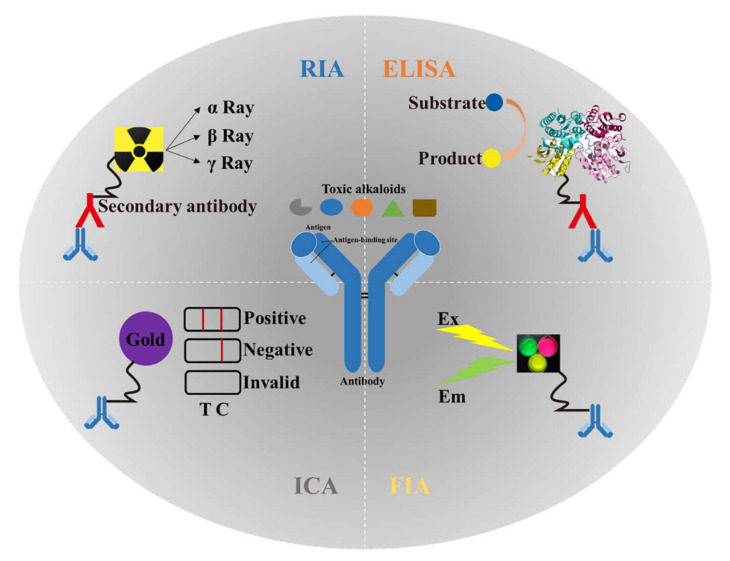
Application and schematic diagram of four types of immunoassays in the detection of plant-derived toxic alkaloids. RIA: radioimmunoassay; ELISA: enzyme-linked immunosorbent assay; ICA: immuno-chromatography assay; FIA: fluoroimmunoassay; Ex: excitation; Em: emission; T: test line; C: control line.

Immunoassays for toxic alkaloids have gone through four stages: RIA, ELISA, ICA, and FIA. The schematic diagram of each type of detection is shown in Figure 3. Among these immunoassays, ELISA and ICA are the two most frequently used methods in the routine analysis of toxic alkaloids. A list of immunoassays used for the analysis of toxic alkaloids is shown in Table 3. Each method includes the reference to the original description, the target analyte, the applicable sample matrices, the sample preparation, and the limit of detection (LOD).

### 4.1. Radioimmunoassay (RIA)

RIA is a new technique for the determination of ultramicro substances in vitro, which combines the high sensitivity, accuracy, and specificity of radioisotope measurement with the specificity of antigen-antibody reaction. In the initial stage of the development of immunoassays, this method was used for the detection of toxic alkaloids, such as morphine [67], vinblastine [89], atropine [97], solasodine [117], and cocaine [118]. Although the method has the advantages of high sensitivity, strong specificity, simple operation, and small sample consumption, the radiation and pollution problems in the analysis process limit the application and development of this method in the detection of toxic alkaloids in plant-derived medicines.

In RIA, the amounts of antibodies are small, and the radioelement labeled antigen competes with the substance to bind to a limited number of antibodies. The sensitivity of the method is high, and the specificity depends on the cross-reactivity of the antibody.

### 4.2. Enzyme-Linked Immunosorbent Assay (ELISA)

ELISA is an immunoassay method using biological enzymes as markers, combining the specificity of the antigen-antibody reaction with the highly efficient catalytic amplification of enzyme. It is a common analytical biochemistry immunoassay, and also the most widely used method in the field of an immunoassay for toxic alkaloids in plant-derived medicines. Unlike macromolecules containing multiple epitopes, toxic alkaloids are generally detected by competitive ELISA because there is only one characteristic functional group of toxic alkaloids that can be recognized by antibodies.

In ELISA, the selectivity and specificity of the method depend on the antibodies. As the reaction in the buffer, antibodies and antigens can compete and combine under the best conditions, and the recognition regions of antibodies in the buffer will not be affected by the coupling methods. Therefore, the sensitivity of this method is high. In addition, the sensitivity of this method is also affected by some reaction conditions such as enzyme quality, substrate selection, etc. These conditions need to be optimized to improve the detection performance of the method.

### 4.3. Immunochromatography Assay (ICA)

ICA is an immunoassay method based on the reversible reaction of antigens and antibodies labeled with gold nanoparticles as tracers. Yusalul et al. (2019) established an ICA method based on the high sensitivity and specificity antibody of monocrotaline, which is time-saving and 16 times more sensitive than ELISA. This method also has many other advantages, such as convenience, low cost, no need for special instruments and pieces of equipment, accurate results, high sensitivity, point-of-care detection, etc., so it develops rapidly. However, there are also shortcomings such as a narrow linear range and insufficient system stability. Therefore, it is necessary to repeat experiments to investigate the influencing factors. This method may also be the most promising tool for the rapid preliminary screening analysis for toxic alkaloids in the future.

In ICA, the interaction between the tracers and antibodies is electrostatic adsorption, and few antibodies are effectively coupled. Thus, the sensitivity is low. Generally, the sensitivity is improved by increasing the number of antibodies within a certain range. Furthermore, it is easy to produce non-specific adsorption, and the cross-reaction rate of some compounds may be increased, resulting in interference of selectivity.

### 4.4. Fluoroimmunoassay (FIA)

FIA is a method that combines the specificity of the immunological response and the sensitivity of the fluorescence technique. Fluorescent nanomaterials have been widely used in clinical diagnosis and imaging due to their broad-spectrum, low toxicity, and strong versatility. In recent years, there have been few reports on the application of fluorescence immunoassays in the detection of toxic alkaloids. The sensitivity of detection can be improved when it is used to label antigens or antibodies. Zheng et al. (2021) prepared fluorescent gold nanoclusters, conjugated with mAb as a label probe, and used in establishing a qualitative and quantitative lateral flow immunoassay for the determination of four pyrrolizidine alkaloids (retrorsine, platyphylline, senecionine, integerrimine) in honey. The LOD was 0.083 μg/kg, and the sensitivity was increased over ten times that of ELISA. Zhang et al. (2020) developed a sensitive, environmental-friendly carbon quantum dots-based FIA for the detection of morphine. It can be applied as a sensitive and convenient tool for the rapid detection of morphine. There are also preliminary studies on colchicine [111]. Given the abundance and diversity of fluorescent nanomaterials, FIA will be of interest to researchers in the future.

FIA has higher sensitivity than the above methods, which depend on the unique properties of the fluorescent materials. However, FIA is less stable than IAC and ELISA, so it is necessary to optimize the reaction conditions such as probe dosage, antibody dosage, buffer solution, pH, and so on to achieve stable and efficient detection performance. Its specificity and selectivity also depend on antibody performance. However, some fluorescent molecules can also recognize non-target objects, leading to non-specific adsorption in the detection process, resulting in false results.

## 5. Prospect

Plant-derived medicines have a long history in the prevention and treatment of human diseases. Now, in the prevention and control of COVID-19, plant-derived medicines have the efficacy of clearing away heat and detoxification, invigorating qi and promoting blood circulation, purifying the lung, and regulating immune function [119]. Toxic alkaloids are one of the important ingredients of plant-derived medicines, and even some of them are the main sources of their medicinal ingredients. Thus, there is a great demand for developing inexpensive and reliable assay methods for effective monitoring of toxic alkaloids to ensure the safe and rational use of plant-derived medicines.

Compared with the traditional chromatographic analysis methods for toxic alkaloids, such as TLC, LC-MS, or GC-MS, immunoassays have inevitable disadvantages. Antibodies are the core reagent of immunoassays. They need a long time to prepare for specificity and need proper storage conditions, which limit the rapid development of immunoassays. Even so, immunoassays have the significant advantages of being fast, relatively inexpensive, and robust enough for in-field measurements, without expensive special personnel for analysis and maintenance. Due to the complex structure and large quantity of plant-derived alkaloids, it is difficult to obtain sufficient standard toxic alkaloids as reference calibrants. Immunoassays could achieve the high-throughput analysis of toxic alkaloids and their structural analogs based on the broad-spectrum antibodies. Besides, the antibodies used in immunoassays can be combined with a variety of nanomaterials or biological probes to achieve a significant increase in sensitivity. Thus, immunoassays can provide strong support for rapid and effective monitoring of toxic alkaloids.

To overcome the existing deficiencies, more in-depth and multifaceted studies in antigen-antibody-based immunoassays are needed. The affinity of the antigen and antibody is the premise of the immunoassay. Much effort has been invested in the development of antibodies against various toxic alkaloids. The antibodies currently developed only specifically identify or cross-identify a small number of highly similar compounds but fail to effectively achieve a high-throughput analysis. Properties of small molecules will influence the antibody response, such as the degree of molecular hydrophobicity, spatial conformation, electron distribution, and physical parameters, which have been researched in antibacterial synergists [120] and veterinary drugs [121]. Modeling the antibody structure and genetic modification of the binding site with site-directed mutagenesis is also a good strategy. Besides, the study of the interaction between the antigen and antibody by molecular modeling and a quantitative structure-activity relationship analysis can reverse guide the design and synthesis of haptens. The research has been conducted on phenylurea herbicides [122] and sulfonamides [123]. Most traditional antibodies used in immunoassays are polyclonal or monoclonal antibodies obtained from immunized mice or rabbits. In recent years, antibodies such as scFv, antigen-binding fragment, or nanobodies obtained from non-immunized animals have attracted more and more attention and have the potential to replace traditional antibodies used in immunoassays. The sensitivity and specificity of antibodies are the key factors that determine the linear range of detection. In addition to antibodies, another problem that restricts the application of immunoassays for toxic alkaloids’ detection is the probe. In recent years, researchers have focused on the development of different types of nanoparticle labels to be used instead of traditionally labeled probes such as enzymes or colloidal gold. These labels include fluorescent probe modified AuNCs, colloidal carbon, and new fluorescence proteins that improve the sensitivity of immunoassays [124,125]. The non-covalent binding force of the antigen and antibody can be detected by amplification of other signals such as electrical signals or fluorescence signals. Most of these new nano-sized labels have not yet been used to develop immunoassays for the detection of toxic alkaloids. Metal-organic frameworks (MOF) have the characteristics of porosity and a large specific surface area, which can be modified with organic ligands to realize the adsorption of a class of compounds with similar properties. This may be an effective method for sample preparation in the future, especially for the extraction of a class of toxic alkaloids with the same properties. In summary, it is necessary to carry out more studies in the field of materials and immune analysis models to optimize new strategies, achieve high-throughput detection of multiple residues of toxic alkaloids, and accelerate the transformation from laboratory technology to real-time, portable, and intelligent field technology, to meet the growing demand for safety monitoring of plant-derived medicines.

## Figures and Tables

**Table 1:** The common toxic alkaloids in plant-derived medicines.

No.	Toxic Alkaloid	CAS	Structure	Classification	Source	Injury Portion	LD_50_	
							Rat	Mouse
1	Aconitine	302-27-2	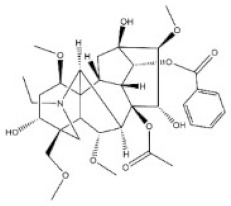	Diterpenoid alkaloid	Ranunculaceae, *Aconitum carmichaeli Debx.*, root	Nervous system	80 μg/kg ^d^	1 mg/mL ^a^; 270 μg/kg ^b^; 270 μg/kg ^c^; 120 μg/kg ^d^
2	Cocaine	50-36-2	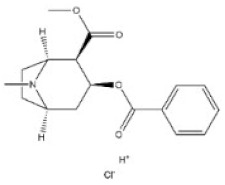	Tropine alkaloid	Erythroxylaceae, *Erythroxylum coca Lam.*, leaf	Nervous system	70 mg/kg ^b^; 250 mg/kg ^c^; 17,500 μg/kg ^d^	99 mg/kg ^a^; 59 mg/kg ^b^; 81 mg/kg ^c^; 16 mg/kg ^d^
3	Scopolamine	51-34-3	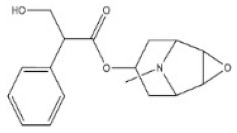	Tropine alkaloid	Solanaceae, *Hyoscyamus niger* L., seed	Respiratory system	2650 mg/kg ^a^	1275 mg/kg ^a^; 400 mg/kg ^b^; 1700 mg/kg ^c^; 100 mg/kg ^d^
4	Atropine	51-55-8	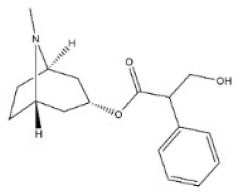	Tropine alkaloid	Solanaceae, *Hyoscyamus niger* L., root, leaf	Nervous system	500 mg/kg ^a^; 280 mg/kg ^b^; 250 mg/kg ^c^; 73 mg/kg ^d^; 920 mg/kg ^e^	75 mg/kg^a^; 30 mg/kg ^b^; 428 mg/kg ^c^; 30 mg/kg ^d^; 550 mg/kg ^e^
5	Colchicine	64-86-8	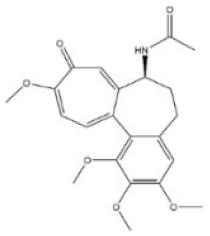	Organic amine alkaloid	Liliaceae, *Iphigenia indica Kunth.*, seed, flower	Respiratory system	1600 μg/kg ^d^;	5886 μg/kg ^a^; 1600 μg/kg ^b^; 1200 μg/kg ^c^; 1700 μg/kg ^d^; 1197 μg/kg ^e^
6	Ephedrine	299-42-3	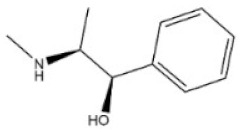	Organic amine alkaloids	Ephedraceae, *Ephedra sinica Stapf.*, stems	Nervous system	600 mg/kg ^a^; 150 mg/kg ^b^; 300 mg/kg ^c^	689 mg/kg ^a^; 350 mg/kg ^b^ 132 mg/kg ^c^; 74 mg/kg ^d^; 170 mg/kg ^f^
7	Strychnine	57-24-9	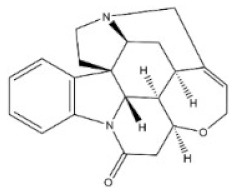	Monoterpenoid indole alkaloid	Loganiaceae, *Strychnos nuxvomica* L., seed	Nervous system	2350 μg/kg ^a^; 1100 μg/kg ^b^; 1200 μg/kg ^c^; 582 μg/kg ^d^; 1400 μg/kg ^e^	2 mg/kg ^a^; 980 μg/kg ^b^; 474 μg/kg ^c^; 410μg/kg ^d^; 628 μg/kg ^e^ 1060 μg/kg ^f^
8	Brucine	357-57-3	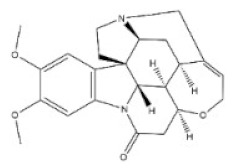	Monoterpenoid indole alkaloid	Loganiaceae, *Strychnos nuxvomica* L., seed	Nervous system	91 mg/kg ^b^	150 mg/kg ^a^; 62 mg/kg ^b^; 60 mg/kg ^c^; 12 mg/kg ^d^
9	Evodiamine	518-17-2	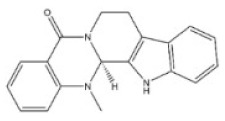	Monoterpenoid indole alkaloid	Rutaceae, *Evodia rutaecarpa (Juss.) Benth.*, mellow fruit	Liver, kidney	-	-
10	Camptothecin	7689-03-4	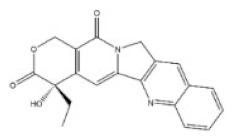	Monoterpenoid indole alkaloid	Nyssaceae, *Camptotheca acuminata Decne.*, root, fruit, bark, branch, leaf	Intestine	-	50,100 μg/kg ^a^; 64 mg/kg ^b^; 38 mg/kg ^d^;
11	Morphine	57-27-2	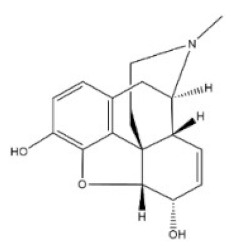	Benzylisoquinoline alkaloid	Papaveraceae, *Papaver somniferum* L., seed	Respiratory system	335 mg/kg ^a^; 100 mg/kg ^b^; 109 mg/kg ^c^; 140 mg/kg ^d^	524 mg/kg ^a^; 140 mg/kg ^b^; 220 mg/kg ^c^; 135 mg/kg ^d^; 6900 μg/kg ^e^
12	Codeine	76-57-3	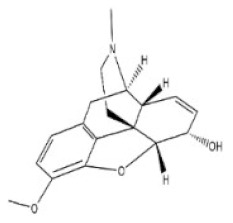	Benzylisoquinoline alkaloid	Papaveraceae, *Papaver somniferum* L., fruit	Respiratory system	427 mg/kg ^a^; 100 mg/kg ^b^; 229 mg/kg ^c^; 75 mg/kg ^d^	250 mg/kg ^a^; 60 mg/kg ^b^; 84,100 μg/kg ^c^; 54 mg/kg ^d^; 290 mg/kg ^e^
13	Berberine	2086-83-1	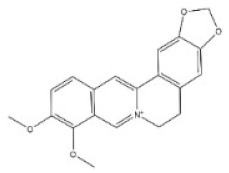	Benzylisoquinoline alkaloid	Rutaceae, *Phellodendron amurense Rupr.*, bark	Heart	-	329 mg/kg ^a^; 18 mg/kg ^c^;
14	Papaverine	58-74-2	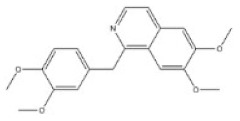	Quinoline alkaloid	Papaveraceae, *Papaver somniferum* L., seed	Nervous system, heart, liver	325 mg/kg ^a^; 59,600 μg/kg ^b^; 151 mg/kg ^c^; 13,300 μg/kg ^d^;	162 mg/kg ^a^; 91 mg/kg ^b^; 170 mg/kg ^c^; 25 mg/kg ^d^;
15	Salsolidine	493-48-1	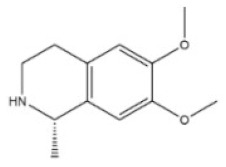	Isoquinoline alkaloid	Chenopodiaceae, *Salsola collina Pall.*, stem, leave	-	-	250 mg/kg ^b^; 170 mg/kg ^d^;
16	Matrine	519-02-8	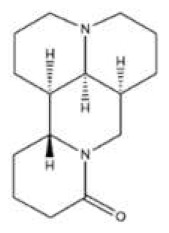	Quinolizidine alkaloid	Leguminosae, *Sophora flavescens Ait.*, root	Nervous system, respiratory system	125 mg/kg ^b^;	150 mg/kg ^b^; 64,850 μg/kg ^d^; 74,150 μg/kg ^e^
17	Retrorsine	480-54-6	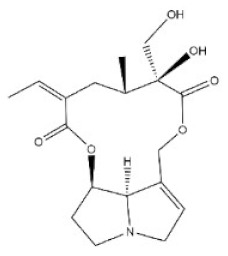	Pyrrolizidine alkaloid	Compositae, *Senecio scandens Buch-Ham.*, the whole herb	Liver, kidney	34 mg/kg ^a^; 34 mg/kg ^b^; 38 mg/kg ^d^	65 mg/kg ^b^; 59 mg/kg ^d^
18	Monocrotaline	315-22-0	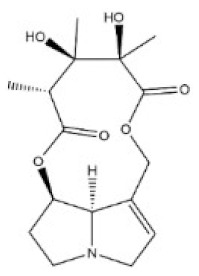	Pyrrolizidine alkaloid	Liliaceae, *Lilium brownie F.E.Br.ex Miellez*, the whole herb	Liver, lung	66 mg/kg ^a^; 60 mg/kg ^c^; 92 mg/kg ^d^	259 mg/kg ^b^; 261 mg/kg ^d^
19	Dichroine	24159-07-7	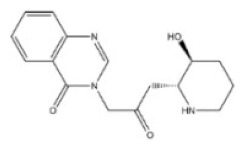	Quinazolidone alkaloid	Saxifragaceae, *Dichroa febrifuga Lour.*, root, leaf	Intestine	-	2500 μg/kg ^a^; 4600 μg/kg ^d^
20	Wilfordine	37239-51-3	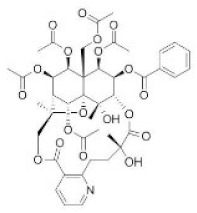	Macrocyclic alkaloid	Celastraceae, *Tripterygium wilfordii Hook. f.*, root, leaf, flower, fruit	Intestine, nervous system	-	-
21	Vincristine	57-22-7	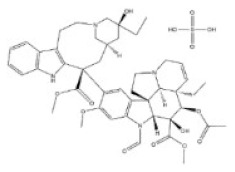	Bisindole alkaloids	Apocynaceae, *Catharathus roseus* (L.) *G.Don*, the whole herb	Intestine, nervous system	1250 μg/kg ^b^; 1 mg/kg ^d^	1300 μg/kg ^b^; 3990 μg/kg ^d^
22	Solasonine	19121-58-5	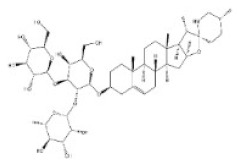	Steroidal alkaloid	Solanaceae, *Solanum nigrum* L., the whole herb	Hemolysis	1500 mg/kg ^b^	77,300 μg/kg ^b^
23	Cytisine	485-35-8	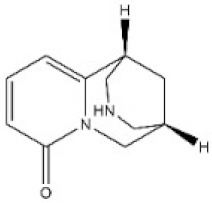	Piperidine alkaloid	Leguminosae, *Sophora tonkinensis Gapnep.*, root, stem	Liver, nervous system, respiratory system, cardiovascular system	8750 μg/kg ^c^	101 mg/kg ^a^; 8550 μg/kg ^b^; 11,764 μg/kg ^c^; 1730 μg/kg ^d^
24	Dauricine	524-17-4	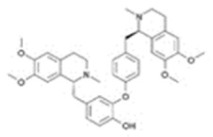	Isoquinoline alkaloid	Menispermaceae, *Menispermum dauricum DC.*, stem	-	-	185 mg/kg ^b^; 1180 mg/kg ^a^
25	Chelidonine	476-32-4	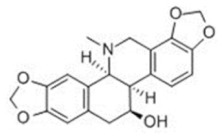	Isoquinoline alkaloid	Papaveraceae, *Chelidonium majus*, the whole herb	-	-	125 mg/kg ^c^; 35 mg/kg ^d^
26	Kumujian A	72755-19-2	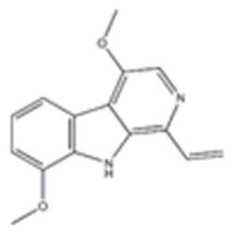	β-Carboline alkaloid	Simaroubaceae, *Picrasma quassioides (D. Don)Benn.*, branch and leaf	-	-	-
27	Conine	458-88-8	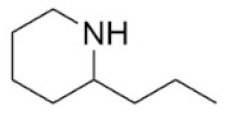	Piperidine alkaloid	Apiaceae, *Cicuta virosa* L.	-	-	100 mg/kg ^a^; 80 mg/kg ^c^; 19 mg/kg ^d^
28	Harmine	442-51-3	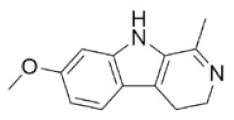	Pyrrolidine alkaloid	Zygophyllaceae, *Peganum harmala* L. the whole herb	Nervous system	200 mg/kg ^c^	243 mg/kg ^c^
29	Ibogaine	83-74-9	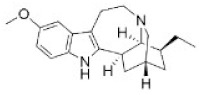	Indole alkaloid	Apocynaceae, *Tabernanthe iboga*, roots, bark, and leaves	Nervous system	327 mg/kg ^a^; 145 mg/kg ^b^	-

a: oral; b: intraperitoneal; c: subcutaneous; d: intravenous; e: intramuscular; f: parenteral; -:no data; The data for LD_50_ (median lethal dose) came from the Chemical Toxicity Database (https://www.drugfuture.com/toxic/, accessed on 31 January 2022).

**Table 2:** The reported antigen and antibody of toxic alkaloids.

Toxic Alkaloid	Synthesis of Antigen		Property of Antibody			Reference
	Hapten	Coupling Method	Type	IC_50_	CR, %	
Aconitine	3-Succinic anhydride aconitine	Active ester method	mAb	-	Aconitine 100.0 Mesaconitine 93.1 Hypaconitine 104.0 Jesaconitine 65.6 Benzoylaconine 8.8 Benzoylmesaconine 5.0 Benzoylhypaconine 1.1	[52]
	3-Glutaric anhydride aconitine		pAb	-	Aconitine 100.0	[83]
	8-Succinic acid aconitine	Single displacement reaction	pAb	-	-	[74]
	8-Hexadecanedioic acid aconitine					
Mono ester aconitine	3-Glutaryl benzoylmesaconine	Active ester method	mAb	1 ng/ml	Benzoylhypaconine 140.0	[92]
Double ester aconitine	Double ester aconitine		mAb	30 ng/mL	Double ester aconitine 100.0	[93]
Cocaine	Benzoylecgonine	Active ester method	mAb	- - -	Cocaine 100.0 Benzoylecgonine 2.2 Norcocaine 13.0	[94]
			mAb	15 nM 180 nM 3100 nM	Cocaine 100.0 Benzoylecgonine 8.3 Ecgonine methylester 0.5	[95]
Higenamine	-	Carbodiimide method	mAb	100 ng/mL	Norlaudanosoline 223.0 (S)-Higenamine 100.0 Tetrahydrobenzyl isoquinoline 4.2 Berberine 3.5	[96]
Cocaine /benzoylecgonine	Diazotized 4-aminococaine/4-aminobenzoylecgonine	Diazo method	mAb	0.4 ng/mL	Cocaine 100.0	[85]
Scopolamine	Nor-scopolamine-N-β-propionic acid	Active ester method	mAb	- - - - -	Scopolamine 100.0 Nor-scopolamine 86.0 Dehydrohyoscyamine 0.7 L-Hyoscyamine 0.2 6-Hydroxy-hyoscyamine 0.2	[80]
Atropine	3-Succinic anhydride tropine	Active ester method	mAb	0.05 ng/mL 0.07 ng/mL 0.14 ng/mL 0.14 ng/mL 0.24 ng/mL 5.30 ng/mL 10.15 ng/mL	Atropine 100.0 Scopolamine 71.4 Homatropine 35.7 Apoatropine 35.7 Anisodamine 20.8 Anisodine 0.9 L-hyoscyamine 0.5	[56]
	Diarotized β-aminobenroic acid L-hyoscyamine	Diazo method	pAb	-	Atropine 100.0	[59]
	3-Succinic anhydride atropine	Active ester method	pAb	6.25 ng/mL	Atropine 100.0	[97]
Colchicine	Colchicine	Carbodiimide method	mAb	3.6 nM	Colchicine 100.0	[70]
Colchicine	Colchicine	-	pAb	-	Colchicine 100.0 3-demethylcolchicine 80.0	[98]
Ephedrine	β-amino-glutaraldehyde methamphetamine	Active ester method	mAb	- - -	Methamphetamine 100.0. Methoxyphenamine 8.0 Ephedrine 2.0	[72]
Camptothecin	20(s)-O-succinyl camptothecin	Active ester method Mixed anhydride method	mAb	2.19 μg/mL 2.85 μg/mL 0.68 μg/mL 0.87 μg/mL	Camptothecin 100.0 Irinotecan 76.6 Topotecan 321.3 Belotecan 250.8	[53]
	Camptothecin 7-carboxylic acid					
Morphine /codeine	Heroin/morphine surrogate	Maleimide-thiol chemistry	-	-	-	[99]
	Acidic derivative of monoacetyl morphine	Active ester method	pAb	0.012 ng/mL 0.01 ng/mL 0.013 ng/mL 0.014 ng/mL	Morphine 100.0 Heroin 120.0 Monoacetylmorphine 92.3 Codeine 85.7	[64]
	Morphine-3-glucuronide		scFv	381.5 pg/mL	Morhpine 100.0	[100]
			pAb	- - - - -	Morphine-3-glucuronide 100.0 Morphine 83.0 Codeine 92.0 Nor-codeine 0.8 6-Monoacetyl morphine 97.0	[78]
	C6-hemisuccinated derivative		mAb	- - -	Morhpine 100.0 Codeine 100.0 Apomorphine 16.5	[69]
	Morphine-6-hemisuccinate	Carbodiimide method	-	-	-	[101]
			mAb	400 pg/mL	Morhpine 100.0	[73]
	N-(4-aminobutyl) normorphine		mAb	100 pg/mL	Morhpine 100.0	[102]
	3-O-carboxymethylmorphine		pAb	-	Morhpine 100.0	[67]
Berberine	9-O-carboxymethyl berberrubine	Active ester method	mAb	12.5 μg/mL 21.9 μg/mL 104.1 μg/mL 8.8 μg/mL 83.3 μg/mL	Berberine 100.0 Palmatine 50.7 9-Acetylberberine 12.3 Coptisine 140.7 Berberrubine 15.1	[90]
Salsolidine	Salsolinol-1-carboxylic acid Salsoline-1-carboxylic acid	Reductive amination	pAb	0.21 pmol	Salsolidine 100.0	[86]
Pyrrolizidine alkaloids	Succinic anhydride retrorsine-	Active ester method	mAb	0.86 ng/mL 0.75 ng/mL 0.43 ng/mL 0.59 ng/mL 19.32 ng/mL 56.32 ng/mL 129.34 ng/mL 184.61 ng/mL 676.45 ng/mL	Retrorsine 100.0 Platyphylline 114.7 Senecionine 200.0 Integerrimine 145.8 Rosmarinine 4.5 Senecivernine 1.5 Neoplatyphylline 0.6 Gynuramine 0.5 Monocrotaline 0.1	[55]
	Succinimid monocrotaline Succinimid retronecine		pAb	0.9 ng/mL 760 ng/mL 100 ng/mL 1 ng/mL	Retrorsine 100.0. Monocrotaline 0.1 Senecionine 0.1 Isatidine (retrorsine N-oxide) 0.9	[76]
	Retrorsine		pAb	23 pg/mL	Senecionine 100.0 Seneciphylline 3.6–34.5	[103]
Vinblastine /vincristine	Diazo vincristine	Carbodiimide method	pAb	1 μg/mL	Vincristine 100.0	[88]
	4-Deacetyl vinblastine C-3 carboxazide	Carbodiimide method	pAb	0.33 ng	Vincristine 100.0	[104]
	N-methyl vinblastine N-formyl vincristine	Mannich reaction	pAb	3.8 μg/mL 2.1 μg/mL	Vinblastine 100.0 Vincristine 55.3	[89]

IC_50_: half maximal inhibitory concentration; CR: cross-reactivity; scFv: single-chain antigen-binding protein; mAb: monoclonal antibody; pAb: polyclonal antibody; -: no data.

**Table 3:** The reported immunoassays of toxic alkaloids.

Target	Method	Sample	LOD	Reference
Aconitine	ICA	Fuzi Lizhong pills	100 ng/mL	[105]
Benzoylhypaconine	ELISA	Rat serum	0.35 ng/mL	[92]
Diester alkaloids	ELISA	Aconitum carmichaeli Debx.	250 pg/mL	[38]
Aco-type alkaloids	ELISA	Aconiti radixes	100 ng/L	[52]
Cocaine	ELISA	Banknotes	5.6 ng/L	[94]
	FIA	-	20 μg/L	[106]
	cEIA	Banknotes and latent fingermarks	0.162 ng/mL	[107]
	ICA	Water	0.26 μg/L	[108]
	ICA	-	0.38 nM	[109]
	RIA	Hair	0.5 ng/mL	[87]
Scopolamine		Plant extract	0.1 ng/mL	[80]
	RIA	*Datura* plants	200 pg	[110]
	ELISA	Hairy root cultures of a Duboisia hybrid	0.2 ng/mL	[61]
Atropine	ELISA	Pig urine, pork, and cereal flour	0.18 ng/mL	[31]
	RIA	Human serum albumin	2.5 ng/mL	[59]
Colchicine	FPIA	Anti-gout pharmaceuticals, milk, and urine	1.8 ng/mL	[111]
	RIA	Plasma	0.2 ng/ml	[71]
Brucine	CL	Urine	2 ng/mL	[112]
Camptothecin	ELISA	-	0.39 ng/mL	[53]
Morphine	ICA	Urine	0.6 ng/mL	[113]
	ICA	Urine	2.5 ng/mL	[99]
	ICA	Urine	0.01 ng/ml	[114]
	ELISA	Urine	1.2 × 10^−11^ M	[79]
	ELISA	Saliva	6 ng/mL	[100]
	RIA	Serum	1 nM	[115]
	RIA	Plasma and brain	-	[67]
Morphine-3-glucuronide	ELISA	Urine	762 pg/mL	[78]
Berberine	ELISA	Plant	780 ng/mL	[90]
Salsolidine	RIA	Serum	0.27 pM	[86]
Retrorsine	ICA	Honey	0.083 μg/kg	[55]
	ELISA	-	0.5 μg/mL	[76]
Monocrotaline	ICA	Plant	0.61 ng/mL	[75]
Triptolide	ICA	Plant	1 μg/mL	[116]
Vincristine	ELISA	-	5 fM	[88]
	RIA	Biological fluids	0.375 nM	[104]
	RIA	Plasma	-	[89]

LOD: the limit of detection; cEIA: competitive enzyme immunoassay; RIA: radioimmunoassay; ELISA: enzyme-linked immunosorbent assay; ICA: immunochromatography assay; FIA: fluoroimmunoassay; -: no data.
